# Survival outcomes of neoadjuvant chemoradiation followed by surgery versus definitive chemoradiation in ESCC: real-world data

**DOI:** 10.3389/fonc.2025.1658595

**Published:** 2025-12-03

**Authors:** Ahmad Alhalabi, Theresa Abdo, Saad Sabbagh, María Herrán, Kaylee Sarna, Rami Tfayli, Zeina Nahleh, Arun Nagarajan

**Affiliations:** 1 Department of Hematology and Oncology, Maroone Cancer Center, Cleveland Clinic Florida, Weston, FL, United States; 2 Center for Clinical Research, Cleveland Clinic Foundation, Cleveland Clinic Florida, Weston, FL, United States

**Keywords:** esophageal cancer, squamous cell carcinoma, neoadjuvant chemoradiotherapy, definitive chemoradiotherapy, real-world data, NCDB

## Abstract

**Background:**

Esophageal squamous cell carcinoma (ESCC) is an aggressive malignancy that has poor survival. Management of ESCC depends on the resectability of the disease, certain patient characteristics, fitness for surgery, and the anatomic complexity of the disease location. Treatment options include either Neoadjuvant chemoradiotherapy (NCR) followed by surgery or definitive Chemoradiation (DCR). While randomized trials have provided insights into both strategies, no large-scale retrospective real-world studies have been conducted to compare these approaches in diverse, unselected populations. This study assesses real-world outcomes of NCR versus DCR using the National Cancer Database (NCDB).

**Methods:**

A retrospective cohort study was conducted using data from the NCDB, focusing on patients with ESCC from 2004 to 2020. Propensity score matching (PSM) (1:1) was utilized. Univariate and multivariate Cox regression analyses were employed.

**Results:**

N = 386 patients with esophageal cancer, with a median age of 64 years (IQR, 57.0-70.0), were identified. Patients were evenly distributed between the DCR and NCR groups, with a total of N = 193 participants. We further selected patients with ESCC, a total of N = 311 patients. NCR demonstrated a significantly extended overall survival (OS) compared to those who received DCR, with a median of 33.15 months vs. 20.5 months (P-value: 0.0069), respectively. Patients receiving DCR had significantly worse OS compared to patients receiving NCR with HR 1.38, 95% CI 1.05–1.81, p=0.0206. Patients with tumors in the thoracic esophagus (C15.3) showed significant survival benefit from surgery (p=0.0070), whereas no benefit was seen for cervical tumors (C15.0), likely due to anatomical complexity and limited sample size.

**Conclusion:**

NCR followed by surgery, when feasible, offers survival benefits for patients with ESCC, especially for locally advanced tumors in the thoracic esophagus.

## 1 Introduction

Esophageal cancer (EC) is an aggressive malignancy and one of the leading causes of cancer-related mortality globally. According to the American Cancer Society, EC accounts for 1% of all cancers diagnosed in the United States (1). The primary histologies for EC include esophageal squamous cell carcinoma (ESCC) and esophageal Adenocarcinoma (EAC), and others. ESCC is typically more common in Asia and parts of Africa, whereas EAC is rising rapidly in Western countries due to lifestyle and metabolic factors (2, 3). Despite advancements in early detection and treatment modalities, the overall prognosis remains poor, especially when diagnosed at an advanced stage. Currently, the mainstay of treatment for EC is multimodal therapy combining chemotherapy, radiotherapy, surgery, and immunotherapy.

For patients with resectable diseases, Neoadjuvant Chemoradiation (NCR) followed by surgery has become the standard of care. Alternatively, Definitive Chemoradiation (DCR) is the treatment of choice for patients who cannot tolerate surgery or have tumors at the proximal or Cervical esophagus, where post-surgical morbidity and mortality is higher (3).

Our study provides contemporary real-world data on ESCC using the National Cancer Database (NCDB), focusing on the comparative survival outcomes of NCR followed by surgery versus DCR alone. We aim to confirm the existing trial-based evidence and explore treatment outcomes based on tumor location, sex, and age. Our findings reinforce the survival benefit of NCR combined with surgical resection in appropriately selected patients, particularly those with locally advanced thoracic esophageal squamous cell carcinoma, to contribute to the ongoing discourse on personalized, multimodal treatment strategies.

## 2 Methods

This retrospective analysis accessed information from the National Cancer Database (NCDB) between 2004 and 2020 for patients with esophageal cancer. The NCDB is a database that compiles data from over 1,500 Commission on Cancer-accredited facilities across the United States. It contains de-identified, HIPAA-compliant patient-level data and is accessible to investigators from accredited programs. Access to such data is only available via an application process and Participant User Data file (PUF) agreement. The study was conducted after obtaining approval by the Cleveland Clinic’s Institutional Review Board (IRB) as Exempt Human Subject Research (IRB #22-153). The waiver of informed consent to participate and publish the data in an online open-access publication was granted in the application due to minimal risk research involving human subjects. This study was conducted per the regulations of the ethical review committee and the guidelines of the journal.

### 2.1 Patient selection

**Figure 1:**
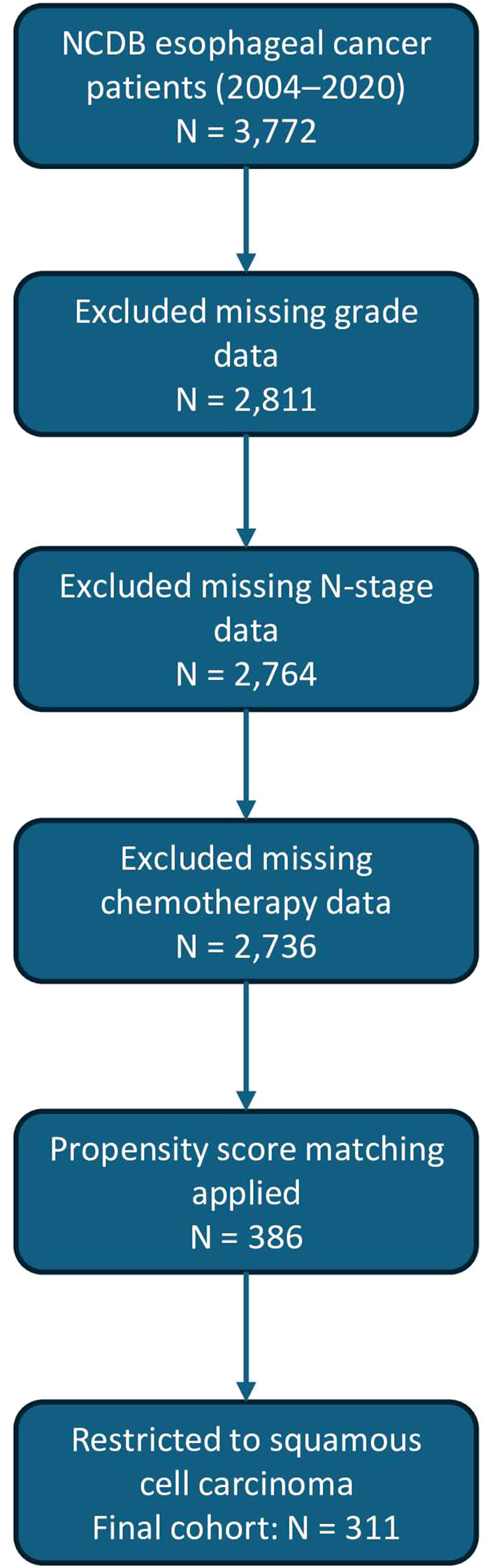
Study Cohort Selection flowchart.

Patients included in this analysis were those diagnosed with esophageal cancer between 2004 and 2020. The data originally included a total of N = 3,772 patients. After excluding patients with missing grade, stage, and chemotherapy data, N = 2,736 patients were included. Patients with missing vital status, which indicates whether the patient is alive or deceased, were excluded from the initial data screening. Propensity score matching (PSM) [1:1] was performed based on the t-stage, n-stage, primary site, Charlson Deyo score, age category, chemotherapy, and grade, yielding a N = 386 patients. We further selected for patients diagnosed with Esophageal Squamous Cell Carcinoma (ESCC), yielding a total N = 311 patients, excluding patients with Adenocarcinoma, Not Otherwise specified, and other diagnoses. Please refer to **Figure 1** that represents the study population flow diagram.

### 2.2 Demographics and clinical variables

Socio-demographic characteristics such as age and sex, clinical-pathological characteristics including primary site, Charlson-Deyo comorbidity score, histology, clinical T stage, grade, clinical N stage, surgery, chemotherapy, regional lymph node surgery, and reason for not receiving radiation, were evaluated in this analysis. Patients were divided into two treatment modality groups: those who received only definitive chemoradiation without surgery (DCR) and those who underwent neoadjuvant chemoradiation (NCR) followed by surgery, esophagectomy. Furthermore, a survival analysis was performed based on age, surgical status, and the primary site. The primary site was identified using the International Classification of Diseases primary site classification “C15.0” for the Cervical Esophagus and “C15.3” for the upper third of the esophagus.

### 2.3 Statistical analysis

Statistical Analysis System (SAS) version 9.4 and R version 4.2.3 were used. PSM 1:1 was performed for the analysis comparing DCR vs. NCR. Surgical procedure of the primary site and reason for no radiation were not included in the propensity score matching because counts couldn’t be matched 1:1. Histology, sex, and regional lymph node surgery weren’t included in propensity score matching to extend sample size and have adequate standardized mean differences. T stage, N stage, primary site, Charlson Deyo score, age category, grade, and chemotherapy were the variables that were matched for propensity score matching. Chi-square, independent t, and Mann-Whitney U tests were performed to evaluate the association between each individual categorical characteristic variable and treatment. P values of 0.05 or less were considered significant. Survival analysis of treatment modalities using Kaplan-Meier curves and multivariable Cox regression were performed.

## 3 Results

### 3.1 Sociodemographic and clinical characteristics

We identified N = 3772 patients with esophageal cancer who were eligible for the analysis. Most patients received DCR as the treatment modality, with N = 3518 (93.3%), while those receiving NCR treatment were N = 254 (6.7%). The pre-propensity score matching demographics and descriptives for patients across different treatment modalities showed statistically significant differences across age, primary site, histology, Charlson Deyo Score, and others. After the 1:1 PSM, we identified N = 386 patients evenly distributed into either DCR or NCR, with N = 193 patients in each group. Our cohort consisted of 63.5% males (n=245) and 36.5% females (n=141), with a median age of 64 years (IQR, 57.0-70.0). Furthermore, more than half of the population had a comorbidity score of zero (68.7%), followed by a score of 1 (26.4%), and scores of 2-3 (4.9%). The Clinical T stages were distributed as follows: T-stage 3 (76.9%), T-stage 2 (15.8%), T-stage 4 (4.9%), and T-stage 1 (2.3%). For the clinical N stage, the distribution was as follows: N-stage 1 (59.8%), N-stage 0 (33.4%), N-stage 2 (5.7%), and N-stage 3 (1.0%). Most patients received multi-agent chemotherapy as their first course of therapy, with N = 341 (88.3%). There was a predominance of squamous cell carcinoma (N = 311, 80.6%) compared to adenocarcinoma (N = 59, 15.3%) (Refer to **Table 1**).

**Table 1:** Chi-square of patients’ baseline sociodemographic and clinical characteristics stratified by treatment modality post PSM.

Characteristics	Total	DCR	NCR	p value
N (%)	386	193	193	
Age, years [median (IQR, Q1, Q3)]	64.0(13.0,57.0,70.0)	65.0(12.0,58.0,70.0)	63.0(14.0,56.0,70.0)	0.3447
Age, years, N (%)				0.9979
18-49	28 (7.3)	14 (7.3)	14 (7.3)	
50-59	93 (24.1)	45 (23.3)	48 (24.9)	
60-69	156 (40.4)	79 (40.9)	77 (39.9)	
70-79	95 (24.6)	48 (24.9)	47 (24.4)	
80-100	14 (3.6)	7 (3.6)	7 (3.6)	
Sex, N (%)				0.3414
Male	245 (63.5)	127 (65.8)	118 (61.1)	
Female	141 (36.5)	66 (34.2)	75 (38.9)	
Primary site, N (%)				1.000
C15.0	32 (8.3)	16 (8.3)	16 (8.3)	
C15.3	354 (91.7)	177 (91.7)	177 (91.7)	
Histology, N(%)				<0.001
Adenocarcinoma	59 (15.3)	9 (4.7)	50 (25.9)	
Squamous cell carcinoma	311 (80.6)	173 (89.6)	138 (71.5)	
Other/NOS	16 (4.1)	11 (5.7)	5 (2.6)	
Grade, N(%)				0.7581
Well differentiated	17 (4.4)	7 (3.6)	10 (5.2)	
Moderately differentiated	230 (59.6)	116 (60.1)	114 (59.1)	
Poorly/undifferentiated	139 (36.0)	70 (36.3)	69 (35.8)	
Charlson Deyo score, N (%)				0.7381
0	265 (68.7)	130 (67.4)	135 (69.9)	
1	102 (26.4)	52 (26.9)	50 (25.9)	
2-3	19 (4.9)	11 (5.7)	8 (4.1)	
Clinical T stage, N (%)				0.9325
1	9 (2.3)	4 (2.1)	5 (2.6)	
2	61 (15.8)	29 (15.0)	32 (16.6)	
3	297 (76.9)	151 (78.2)	146 (75.6)	
4	19 (4.9)	9 (4.7)	10 (5.2)	
Clinical N stage, N (%)				1.000*
0	129 (33.4)	64 (33.2)	65 (33.7)	
1	231 (59.8)	116 (60.1)	115 (59.6)	
2	22 (5.7)	11 (5.7)	11 (5.7)	
3	4 (1.0)	2 (1.0)	2 (1.0)	
Surgery, N (%)				<0.001
Esophagectomy	193 (50.0)	0 (0.0)	193 (100.0)	
No surgery	193 (50.0)	193 (100.0)	0 (0.0)	
Chemotherapy, N (%)				0.8922
chemo given as first course therapy, but type not specified	23 (6.0)	11 (5.7)	12 (6.2)	
Single-agent chemo as first-course therapy	22 (5.7)	12 (6.2)	10 (5.2)	
Multi-agent chemo as first-course therapy	341 (88.3)	170 (88.1)	171 (88.6)	
Regional lymph node surgery, N (%)				<0.001
No regional lymph node surgery	195 (50.5)	187 (96.9)	8 (4.1)	
Regional lymph node surgery	191 (49.5)	6 (3.1)	185 (95.9)	
Reason for no radiation, N (%)				
Radiation therapy administered	386 (100.0)	193 (100.0)	193 (100.0)	—

Bold values are statistically significant.

### 3.2 Survival analysis

**Figure 2:**
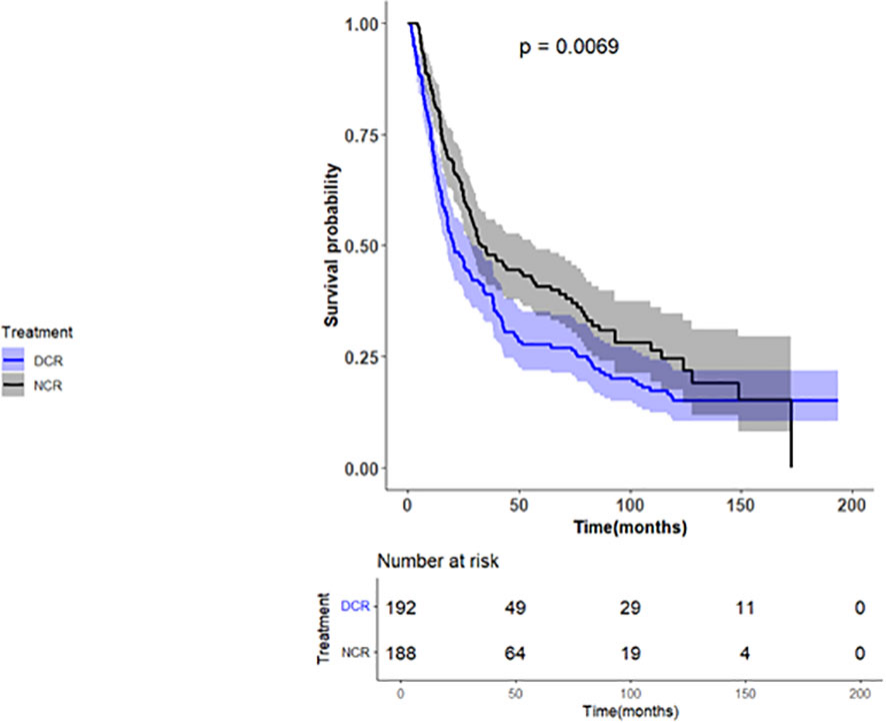
Kaplan–Meier plot of overall survival in patients with esophageal cancer, stratified by neoadjuvant chemoradiation versus definitive chemoradiation.

All variables listed in **Table 1**, except for surgery and the reason for no radiation, were added to the univariate Cox regression analysis. Sex, Charlson Deyo score, regional lymph node surgery, and treatment were significant and were included in the multivariate model. After applying backward elimination, only the regional lymph node surgery (p=0.1622) was removed. Patients treated with NCR had the most extended overall survival (OS), with a median of 33.15 months (95% CI 27.79-55.56), in comparison to those who received DCR, which had a median of 20.5 months (95% CI 17.12-28.12), with a log-rank p-value of 0.0069, as shown in **Figure 2**.

Female patients were less likely to die than males, with a Hazard Ratio (HR) of 0.67 (95% CI 0.5-0.88, p-value=0.0041). Furthermore, patients who underwent DCR had an increased risk of death compared to those who received NCR with a HR of 1.41 (95% CI 1.11-1.79, p-value=0.0045). **Table 2** presents the multivariable Cox regression analysis.

**Table 2:** Multivariate cox regression analysis of clinical and demographic predictors of overall survival.

Variable	HR (95%CI)	p-value
Sex (Overall)		
Male (ref.)	1.00	–
Female	0.68(0.53-0.88)	**0.0033**
Treatment (Overall)		
DCR	1.41(1.11-1.79)	–
NCR (ref.)	1.00	**0.0045**
Age category (ESCC subset)		
18–59 years (ref.)	1.00	–
60+ years	1.15(0.86-1.52)	0.3497
Surgery type (ESCC subset)		
Esophagectomy (ref.)	1.00	–
No surgery	1.42(1.08-1.86)	**0.0125**
Primary site (ESCC subset)		
C153 (ref.)	1.00	–
C150	0.64(0.35-1.19)	0.1605
Surgery type (C15.0 Subset)		
Esophagectomy(ref.)	1.00	–
No surgery	0.68(0.16-2.79)	0.5884
Surgery type (C15.3 Subset)		
Esophagectomy (ref.)	1.00	–
No surgery	1.44(1.09-1.90)	**0.0108**
Charlson Deyo Score (C15.3 subset)		
0 (ref.)	1.00	–
1	1.12(0.80-1.56)	0.5052
2	2.22(1.28-3.85)	**0.0045**

Bold values are statistically significant.

### 3.3 Subset analysis: ESCC

We have performed survival analysis exclusively on patients with Esophageal squamous cell carcinoma. After excluding patients with Adenocarcinoma or other histologies, a total population of N = 311 was obtained. The OS benefit of NCR persisted in this cohort. Patients receiving DCR had a significantly increased risk of death compared to patients receiving NCR, with an HR 1.38 (95% CI 1.05-1.81, p-value = 0.0206). Female patients were still significantly less likely to die compared to male patients with an HR of 0.67 (95% CI 0.5-0.88, p-value=0.0041). Furthermore, the cohort was divided into two strata based on their age. Patients aged between 18 to 59 vs. Patients aged 60 +. No significant survival difference between the two strata, with patients aged 60+ having a higher risk of death with an HR of 1.15 (95% CI 0.86-1.52, p-value=0.3497).

### 3.4 Primary site analysis

**Figure 3:**
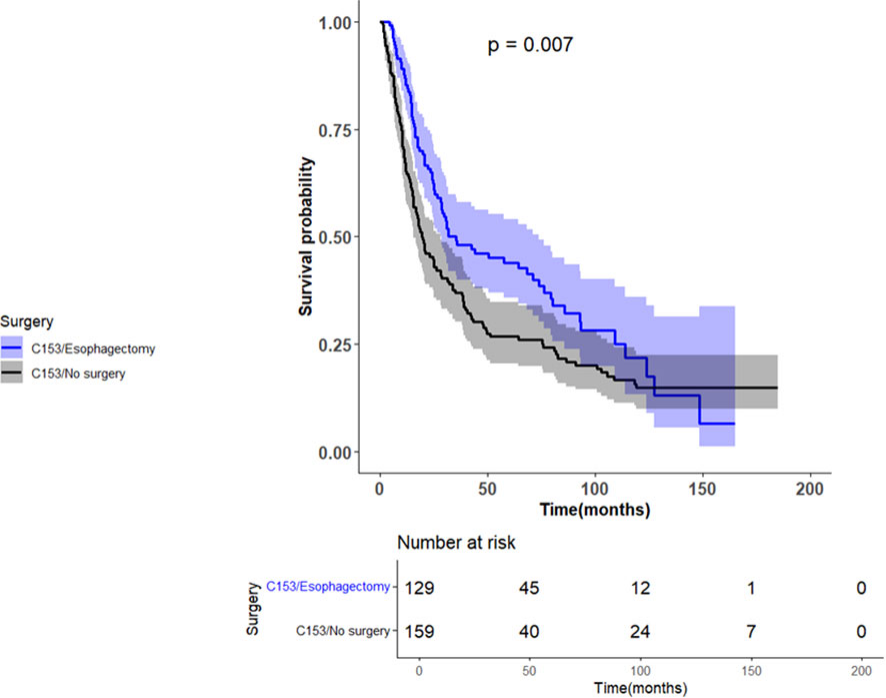
Kaplan–Meier plot showing overall survival among patients with thoracic ESCC (C15.3), stratified by surgery status.

We further stratified the survival analysis based on the tumor location. Patients receiving esophagectomy for esophageal tumors at C15.3 (upper third) were associated with an improved median overall survival compared to no surgery (35.15 vs. 19.38 months; p-value=0.0070). Patients who did not undergo esophagectomy had a significantly increased risk of death with an HR 1.44 (95% CI 1.09-1.90, p-value=0.0108), **Figure 3**. Additionally, a Charlson Deyo-score of 2 was independently associated with worse survival with an HR 2.22 (95% CI 1.28-3.85, p-value= 0.0045).In contrast, patients with esophageal tumors at C15.0 (Cervical esophagus) who did not undergo esophagectomy had an improved chance of survival by 32% compared to patients who received esophagectomy. However, the difference between the two groups was not statistically significant (p-value = 0.5884). Notably, the small sample size (n = 19) limited the power analysis in this subgroup.

## 4 Discussion

To our knowledge, this retrospective cohort analysis is among the largest, matched, real-world study focusing exclusively on ESCC, demonstrating that neoadjuvant chemoradiation followed by surgery confers a survival advantage over definitive chemoradiation, with outcomes further stratified by tumor location. This work confirms and reaffirms the results of existing clinical trials and clinical practices in the management of ESCC.

Neoadjuvant chemoradiation is established standard of care for the management of ESCC (4, 5). Randomized trials such as CROSS and NEOCRTEC5010, along with multiple cohort studies have demonstrated superiority in survival outcomes for patients who receive NCR plus surgery in patients with resectable lower and midthoracic locally advanced ESCC (6–11).The Cross trial reported long-term overall survival rate, improved R0 resection (R0 = clear negative margins), and a pathological complete response (pCR) (6). This survival advantage could be driven by several mechanisms. Neoadjuvant chemoradiation serves as a backbone, allowing for downstaging and reduction of the disease burden. In the CROSS trial, patients who received NCR plus surgery had a significantly higher R0 resection rate (92% vs. 69%, respectively). Tumor downstaging and size reduction are well-established predictors of long-term overall survival (12, 13) Adding surgical intervention would provide a synergistic effect, reducing local recurrence and mitigating the relatively high risk of ESCC locoregional spread through submucosal invasion (14). Furthermore, NCR provides a means to eradicate micro-metastatic disease, reducing early recurrences and improving disease-free survival (15).

NCR and DCR employ similar chemoradiotherapy backbone, with surgery serving as the key differentiating factor. In their meta-analysis, Kamarajah et al. compared NCR followed by surgery to DCR in locally advanced ESCC, reporting improved overall survival with the NCR group (16). The regimen consists of a combination of platinum and fluoropyrimidine (Cisplatin + 5-FU, or variants such as Cisplatin + Capecitabine). Less frequently, it can include docetaxel. Stahl et al., in their meta-analysis, compared radiotherapy dosages between NCR and DCR. NCR utilizes a lower dose of radiotherapy, ranging from 40 to 50.4 Gy, whereas DCR typically uses higher dosages, ranging from 50 to 66 Gy. Although DCR regimens typically use higher radiation doses, evidence of a corresponding survival benefit remains inconsistent, and surgery may contribute meaningfully to long-term disease control (17).

Tumor location is essential in determining the optimal treatment modality. Surgery provided a survival advantage for patients with thoracic esophageal tumors (C15.3), with a median overall survival of 35.15 months compared to 19.38 months (p-value = 0.0070). However, this advantage was not observed in proximal tumors (C15.0). The analysis suggested a survival advantage for patients managed without surgery, HR 0.68 (p-value = 0.5884). The findings must be interpreted with caution given the very small sample size (n=19). These results are expected since tumors located more proximally carry a greater surgical risk. NCCN guidelines and Waters, Reznik, and Deboever’s study highlighted that DCR is the standard of care for cervical and high thoracic ESCC, as surgery would pose a higher risk of morbidity and mortality due to the proximity of vital anatomical structures (7, 18, 19).

Furthermore, our analysis found that female sex was independently associated with a survival advantage consisted with prior reports (20, 21). This may be attributed to hormonal, behavioral, and biological factors (20). Importantly, Kalff et al. in their population-based study explain this interesting observation, attributing it to differences in tumor biology and a usual fewer comorbidity compared to males. Notably, they also highlight the existence of unconscious bias in treatment decisions, whereby under-treatment could mask the true female survival advantage (21).

Moreover, literature exists comparing chemotherapy stand alone vs. Chemoradiation therapy. In the NeoRes-I clinical trial, it compared two neoadjuvant modalities. While CRT was associated with a higher pathological complete response (pCR) rate, no significant differences were observed in overall survival (OS) or progression-free survival (PFS) between the two groups. Notably, the CRT group experienced a higher rate of postoperative mortality (22). This could be due to radiotherapy toxicity, inflammation, and other complications. Wang et al. demonstrated in their review paper how radiotherapy could lead to lymphopenia and adversely affect outcomes. They suggested strategies to mitigate radiation-induced lymphopenia, such as using different techniques and the possibility of using proton beam therapy (23). Similarly, the JCOG1109 (NExT) trial demonstrated that although chemoradiation achieved higher pCR, the triplet chemotherapy regimen, Cisplatin + 5-Fluorouracil (CF) + Docetaxel (D) (NeoCF+D), provided a significantly better 3-year overall survival in locally advanced ESCC patients, particularly in fit populations (24). In contrast, the FFCD 9102 and FFCD 9901 trials showed no OS benefit of surgery over DCR in early-stage disease, NCR did not improve survival in these early-stage cohorts, reinforcing the role of DCR in this specific setting (25, 26). The differences in results may be attributed to variations in tumor histology and disease stage. Our study concentrated on Esophageal squamous cell carcinoma (ESCC), whereas the FFCD trials included mixed histologies. Additionally, we had a cohort consisting of patients with locally advanced thoracic ESCC, which might benefit from a multimodal treatment approach. In contrast, the FFCD trials enrolled patients with early-stage disease. Furthermore, Stahl et al. emphasized that definitive chemoradiotherapy remains a curative option in ESCC, with local control being the primary benefit of adding surgery (27).

It is important to highlight the rapidly evolving role of immunotherapy in the management of esophageal cancer, particularly its integration into the adjuvant setting. Multiple clinical trials have been conducted, and others are ongoing to improve outcomes in patients with esophageal cancer. The CheckMate 577 trial found that adjuvant Nivolumab significantly improves DFS in patients with residual disease after NCR followed by surgery (28). Further analysis has shown that patients with ypN+ disease (pathologically node-positive status after surgery) and PD-L1 combined positive score (CPS ≥5) experienced the most significant benefit. At the same time, outcomes were less favorable in ypN0 (no residual nodal disease) or PD-L1 low subgroups (29). In addition, Panda and Schumacher reinforced the crucial role of lymphadenectomy and accurate staging within the evolving Immuno-Oncology era (30). Multiple trials, such as MATTERHORN, KEYNOTE-585, and ATTRACTION-5, are investigating the benefits of checkpoint inhibitors in the neoadjuvant or perioperative setting (31–33). More studies are shifting towards precision oncology, focusing on minimal residual disease (ctDNA) and incorporating biomarkers (34).

Our work is one of the largest real-world NCDB analyses that compares NCR vs. DCR exclusively in ESCC, providing stratification by tumor localization and sex. This data use propensity score matching and multivariate Cox regression to balance existing confounders. However, we acknowledge the existence of certain limitations inherent in the retrospective design, including potential selection bias, unmeasured confounders, and immortal time bias are possible. NCDB does not provide other metrics of survival, such as performance status, disease progression, recurrence status, and details regarding chemotherapy regimens. Although peri-operative mortality are available, our endpoint was long term overall survival of the two treatment modalities. In the subgroup analysis of C15.0 Cervical ESCC, the cohort is small (N = 19) and should be interpreted with caution. Additionally, NCDB includes de-identified data from community and academic centers with varying levels of surgical expertise and radiation protocols, which may impact generalizability. Despite these limitations, our data is consistent with the results of clinical trials and practices.

## 5 Conclusion

Our findings reaffirm that NCR and surgery, when feasible, offer survival benefits for patients with ESCC, especially for locally advanced tumors in the thoracic esophagus. NCR should remain central in the multidisciplinary management of this aggressive malignancy.

## Data Availability

The datasets presented in this article are not readily available because the data supporting this study’s findings are available for investigators who are granted access to the National Cancer Database through an online application from the ACS website [https://www.facs.org/quality-programs/cancer-programs/national-cancer-database/puf/]. The participant user files (PUF) for the esophageal cancer database were downloaded by the principal investigator associated with a Commission on Cancer (CoC) accredited institution. Requests to access the datasets should be directed to [https://www.facs.org/quality-programs/cancer-programs/national-cancer-database/puf/].
